# Integration analysis of single-cell and spatial transcriptomics reveal the cellular heterogeneity landscape in glioblastoma and establish a polygenic risk model

**DOI:** 10.3389/fonc.2023.1109037

**Published:** 2023-06-15

**Authors:** Yaxuan Liu, Zhenyu Wu, Yueyuan Feng, Jiawei Gao, Bo Wang, Changlin Lian, Bo Diao

**Affiliations:** ^1^ School of Laboratory Medicine and Biotechnology, Southern Medical University, Guangzhou, Guangdong, China; ^2^ Department of Basic Medicine, General Hospital of Central Theatre Command, Wuhan, Hubei, China; ^3^ Department of Urology, The First People’s Hospital of Foshan, Foshan, Guangdong, China; ^4^ Cancer Hospital, The First People's Hospital of Foshan, Foshan, Foshan, Guangdong, China; ^5^ College of Medicine, JiShou University, Xiangxi, Hunan, China; ^6^ Department of Neurosurgery, Wuhan General Hospital of Guangzhou Command and Hubei Key Laboratory of Central Nervous System Tumor and Intervention, Wuhan, Hubei, China

**Keywords:** glioblastoma, tumor microenvironment, ScRNA-seq, spatial transcriptomics, heterogeneity, immune infiltration, tumor progress-related gene risk score

## Abstract

**Background:**

Glioblastoma (GBM) is adults’ most common and fatally malignant brain tumor. The heterogeneity is the leading cause of treatment failure. However, the relationship between cellular heterogeneity, tumor microenvironment, and GBM progression is still elusive.

**Methods:**

Integrated analysis of single-cell RNA sequencing (scRNA-seq) and spatial transcriptome sequencing (stRNA-seq) of GBM were conducted to analyze the spatial tumor microenvironment. We investigated the subpopulation heterogeneity of malignant cells through gene set enrichment analyses, cell communications analyses, and pseudotime analyses. Significantly changed genes of the pseudotime analysis were screened to create a tumor progress-related gene risk score (TPRGRS) using Cox regression algorithms in the bulkRNA-sequencing(bulkRNA-seq) dataset. We combined the TPRGRS and clinical characteristics to predict the prognosis of patients with GBM. Furthermore, functional analysis was applied to uncover the underlying mechanisms of the TPRGRS.

**Results:**

GBM cells were accurately charted to their spatial locations and uncovered their spatial colocalization. The malignant cells were divided into five clusters with transcriptional and functional heterogeneity, including unclassified malignant cells and astrocyte-like, mesenchymal-like, oligodendrocytes-progenitor-like, and neural-progenitor-like malignant cells. Cell-cell communications analysis in scRNA-seq and stRNA-seq identified ligand-receptor pairs of the CXCL, EGF, FGF, and MIF signaling pathways as bridges implying that tumor microenvironment may cause malignant cells’ transcriptomic adaptability and disease progression. Pseudotime analysis showed the differentiation trajectory of GBM cells from proneural to mesenchymal transition and identified genes or pathways that affect cell differentiation. TPRGRS could successfully divide patients with GBM in three datasets into high- and low-risk groups, which was proved to be a prognostic factor independent of routine clinicopathological characteristics. Functional analysis revealed the TPRGRS associated with growth factor binding, cytokine activity, signaling receptor activator activity functions, and oncogenic pathways. Further analysis revealed the association of the TPRGRS with gene mutations and immunity in GBM. Finally, the external datasets and qRT-PCR verified high expressions of the TPRGRS mRNAs in GBM cells.

**Conclusion:**

Our study provides novel insights into heterogeneity in GBM based on scRNA-seq and stRNA-seq data. Moreover, our study proposed a malignant cell transition-based TPRGRS through integrated analysis of bulkRNA-seq and scRNA-seq data, combined with the routine clinicopathological evaluation of tumors, which may provide more personalized drug regimens for GBM patients.

## Introduction

Glioblastoma (GBM), a type IV glioma classified by the World Health Organization, is a highly aggressive brain tumor (1, 2). Among all malignant brain and other central nervous system (CNS) tumors, GBM accounts for 49% of all age groups (3). The incidence of GBM increases with age, with rates highest in individuals between 75 and 84 years of age (3). In North American countries, the combined incidence of malignant brain and other CNS tumors in all age groups has decreased by about 0.8% per year due to increased medical access and public awareness (3–5). Although the last decade of immunotherapy has brought hope to cancer patients, the subpopulation of patients expected to show a response has not been identified. Until recently, there has been a consistent lack of improvement in the 5-year survival rate of elderly patients (3–5). This is due to our poor understanding of the brain’s neuroimmune system and the presence of the blood-brain barrier, making standard therapeutic strategies and immunotherapy less effective in GBM (6, 7). In addition, the complex microenvironment of GBM is one factor that impedes the antitumor treatment and causes tumor recurrence (8). TME comprises tumor cells, stromal cells, signaling molecules, immune cells, and the surrounding extracellular matrix (ECM) (9). It has been reported that GBM is mainly composed of four subtypes of malignant cells, including astrocyte-like (AC-like), mesenchymal-like (MES-like), oligodendrocytes-progenitor-like (OPC-like) and neural-progenitor-like (NPC-like) subtypes (10, 11). Therefore, the heterogeneity of GBM will bring difficulties to clinical treatment. There is an urgent need for predictive biomarker to help us clinically identify subpopulations of GBM patients expected to show response.

The analyses for single-cell RNA sequencing (scRNA-seq) have been widely applied to identify diverse cell types and expand the understanding of their role in brain tumors (12, 13). Furthermore, some studies constructed a risk model of ferroptosis-related genes through scRNA-seq data to predict the overall survival and progression-free survival of GBM patients (14). Risk models are also derived based on the signature of genes involved in angiogenesis, autophagy, apoptosis, and necrosis in glioblastoma (15, 16). These models may guide effective treatment strategies for patients. However, scRNA-seq inherently loses its cellular spatial information during the tissue dissociation step, and conclusions from a single database are not convincing.

In contrast, spatial transcriptome sequencing (stRNA-seq) is limited to measuring spots with mixtures of cells and cannot identify diverse cell types in a single-cell resolution (17). The current studies revealed spatial TME in GBM through integrated analysis of scRNA-seq and stRNA-seq data. The heterogeneity of GBM was explored in scRNA-seq data and stRNA-seq data. Differentiation trajectory analysis of the proneural–mesenchymal transition in GBM and cell-cell communications analysis in the tumor microenvironment indicated that GBM cells, together with the tumor microenvironment, promote malignant cell transcriptomic adaptability and disease progression. Furthermore, we constructed TPRGRS to predict the prognosis of GBM through integrated analysis of bulkRNA-seq data and scRNA-seq data, which may help clinicians provide more personalized treatment.

## Materials and methods

### Dataset collection and preprocessing

The scRNA-seq dataset (GBM_GSE131928_10X) was downloaded from the TISCH (http://tisch.comp-genomics.org/). The stRNA-seq dataset (Human Glioblastoma: Whole Transcriptome Analysis) was downloaded from the 10xGenomics datasets (https://www.10xgenomics.com/). We downloaded bulkRNA-seq profiling information in Transcripts Per Million (TPM) format of 168 GBM patients, as well as the corresponding clinical data from the TCGA database, normalized RNA expression data, and complete clinical data of 147 GBM patients from the CGGA database (18), and RNA expression data and corresponding clinical information of 94 GBM patients from the REMBRANDT database (19). The batch effect of bulkRNA-seq data was adjusted through the “sva” R package.

### Single-cell sequencing and spatial transcriptomics data processing

We used the R package Seurat (v4.1.0) to process single-cell transcriptomics and spatial transcriptomics data [11]. We first used the “Read10X_h5” function to read the cell matrix profile downloaded from the TISCH website. Then, the function “CreateSeuratObject” was applied to convert the matrix into a Seurat object. We excluded those cells with fewer than 500 genes, more than 4,000 genes, or more than 15% mitochondria content. We log-normalized the Seurat object and identified highly variable features using the “FindVariableFeatures” function with the parameters selection(method = vst, and nfeatures = 2000. We subsequently scaled the seurat object and performed linear dimensional reduction using the RunPCA function with the variable features of the seurat object. We visualized the distribution of each principal component using the “ElbowPlot” function and used the first 15 principal components for clustering. We performed K-nearest neighbors clustering for the seurat object with the parameter dims = 1:15 through “FindNeighbors” function. We performed FindClusters function with the parameter resolution =0.5, and Uniform Manifold Approximation and Projection (UMAP) clustering using the RunUMAP function with the parameter dims = 1:15.We then identified the cell type of each cell cluster according to the cell information of TISCH. For spatial transcriptomics of GBM, we applied the function “SCTransform” to normalize the data of spatial transcriptomics. We used functions “RunPCA”, “FindNeighbors” and “FindClusters” to reduce the dimensionality and cluster similar spatial spots.

### Spatial map of cell types in glioblastoma

Different spots of stRNAseq were preliminarily annotated based on the CELLTREK toolkit (version 0.0.94), which provided a more flexible and direct way to investigate spatial transcriptomics at a single-cell resolution (20). CELLTREK directly maps cells back to spots in GBM spatial transcriptomics by co-embedding the processed GBM single-cell transcriptomics and spatial transcriptomics. First, we co-embed stRNA-seq and scRNA-seq datasets using “train”. After embedding, we chart cell types of scRNA-seq data to their spatial locations through the function “celltrek” (**Supplementary Figure S1**). Then, we visualized the CellTrek results and directly investigated the stRNA-seq data with spatial topography. Then we used the function “SColoc” to perform colocalization analysis and visualize the colocalization result.

### Subclustering of the malignant cell clusters

The procedure of subclustering the malignant cell cluster was the same as that of the pan-cell dataset. CellTrek was applied to identify malignant cell clusters in stRNA-seq data.

### Functional enrichment analysis of malignant cells in GBM

To investigate differences in transcriptome and function between cell types in scRNA-seq and stRNA-seq data, we used the function “FindAllMarkers” of Seurat to find differentially expressed genes (DEGs) of cell types in the GBM, DEGs of each cell type were used for visualization. Then, Gene Set Enrichment Analysis (GSEA) of DEGs was performed to determine the enrichment score of oncogenic hallmark pathways in malignant cells (p. value< 0.05). Differentially expressed genes were also applied in different malignant subtypes (21). Gene Set Variation Analysis (GSVA) was employed to analyze the enrichment results of different malignant cell subtypes. The oncogenic hallmark pathways genesets(h.all.v7.1.symbols) were downloaded from the MSigDB database (22, 23).

### Cell-cell communication in the TME

For the inference and analysis of cell-cell communication between subtypes of malignant cells and non-malignant cells in the TME, CellChat (version 1.1.3) was used to infer the cell-cell interactome by assessing the gene expression of ligand-receptor pairs across cell types in RNA-seq and stRNA-seq (24). The function of “AggregateNet” in CellChat is to aggregate cell-cell communication network involved in nine cell types of GBM, including subtypes of malignant cells (AC-like, MES-like, NPC-like, OPC-like, and unclassified GBM malignant cell), CD8 Tex, M1, Monocyte and Oligodendrocytes. Signaling pathways in cell-cell communication networks were calculated and visualized by the function of “netVisual_aggregate”. Cellchat computed centrality scores and identified major signaling roles of cell types in the TME using the function “netAnalysis_computeCentrality”.

### Pseudotime analysis of malignant cells

We analyzed the trajectory of malignant cells in the scRNA-seq, and pseudotime developmental trajectories were constructed by Monocle2 (version 2.22.0) (25). Then, the hub genes in each subtype of malignant cells were recognized using the function “differential Gene Test” in the monocle2 package. The hub genes were filtered out based on q.values (q< 0.01) to order genes of the developmental trajectory. The “DDRTree” method was used to reduce the dimension of single cells. Single cells were ordered into a trajectory with branch points, multiple branches and nodes were observed throughout the developmental trajectory, and cells on the same branch were considered to have the same state. Pseudotime-related genes were determined using the function of “differentialGeneTest” in the Monocle2 package. The plot genes in pseudotime function to discover transitional changes in gene expression levels along the pseudotime. Different malignant cell subtypes with pseudotime values in scRNA-seq data were mapped to stRNA-seq data through CellTrek. To identify the order in which functional events are acquired or lost during the transition of malignant cells, we used GeneSwitches (version 0.1.0) to process scRNA-seq data together with pseudotime trajectories to order pathways along the pseudotime (26). GeneSwitches filtered genes for pathway analysis throughout pseudotime using the “filter switchgenes”. The function of “find switch pathways” were used to find significantly changed pathways with pseudotime trajectories.

### Generation of the tumor progress-related gene risk score model

To establish the tumor prognosis-related signature, we employed the TCGA dataset as the training set, while the CGGA and REMBRANDT datasets were the validation sets. Univariate Cox analysis was performed to screen for critical genes from the identified malignant cells and pseudotime-related genes associated with the OS of patients with GBM. Random survival forest (RSF) analysis was then conducted using the “Random Forest SRC” R package to narrow the prognostic gene panel further. In RSF analysis, variables were ranked by minimal depth, of which a smaller value indicated greater predictiveness. Next, Multivariate Cox regression analysis was used to establish the optimal tumor prognosis-related signature based on respective coefficients (β) and gene expression levels. This formula calculates each patient’s tumor progress-related gene risk score (TPRGRS). Subsequently, we divided the patients into low- and high-risk groups based on the median TPRGRS. The Kaplan-Meier approach was applied to determine the prognostic difference between the two groups. We further evaluate the correlations between the TPRGRS and clinical features, including age, gender, primary-recurrent-secondary (PRS) type, radiation therapy, chemotherapy, Isocitrate Dehydrogenase (NADP (+)) (IDH) mutation, the short arm of chromosome 1 (1p) and the long arm of chromosome 19 (19q) (1p19q) status, O-6-Methylguanine-DNA Methyltransferase phosphorylation (MGMTp) methylation. Univariate and Multivariate Cox analyses were utilized to assess the prognostic significance of TPRGRS. Meanwhile, we collected the CGGA and REMBRANDT datasets to verify TPRGRS’s predictive efficacy.

### Functional enrichment analysis of bulkRNA-seq

To investigate the underlying mechanism regarding TPRGRS, differentially expressed genes were obtained between the low- and high-risk groups. First, we performed Gene Ontology (GO) enrichment using the “clusterProfiler” R package (27). GO terms with p< 0.05 were visualized by the “circlize” R package. GSVA was employed to determine the differences between the two groups on the oncogenic hallmark pathways (22, 23). GSEA was performed between the two groups for the same hallmark pathways with the “GSEA” R package (FDR< 0.25, and p.adjust< 0.05) (21). Kaplan-Meier method was employed to determine the prognostic significance of oncogenic hallmark pathways.

### Somatic mutation analysis

The somatic mutations of GBM patients were extracted from the TCGA database. The “maftools” R package explored the specific somatic mutation variations in different TPRGRS groups (28). Next, we investigated the mutually co-occurring or exclusive mutations, tumor-causing genes, and enrichment of known oncogenic pathways between the two cohorts.

### Immune landscape analysis and drug prediction

We compared the low- and high-risk groups’ immune cell abundance based on TIMER, CIBERSORT, CIBERSORT-ABS, QUANTISEQ, MCPCOUNTRE, XCELL, and EPIC (29–34). We use the single sample Gene Set Enrichment Analysis (ssGSEA) to compare the low- and high-risk groups’ immune function. Using Spearmen’s correlation test, we explored the expression of immune cell inhibitory receptors and ligands in the low- and high-risk groups. Meanwhile, we searched for chemotherapy drugs from the CGP database. We investigated the chemotherapy response of the two groups, and the “pRRophetic” R package predicted the IC50 values of chemotherapeutic drugs for each patient (35).

### Validation of the TPRGRS

The GEPIA database (http://gepia.cancer-pku.cn/) consists of bulkRNA-seq samples derived from the TCGA, and GTEx database was used to verify the gene expression level of TPRGRS (36). *P*<0.05 was considered to be statistically significant. We applied Quantitative Real-Time Polymerase Chain Reaction (qRT-PCR) to validate the gene expression level of TPRGRS in tumor and normal cells. Two glioblastoma cell lines (U251 and U87) and one normal human astrocytes cell line (NHA) were purchased from Procell (Wuhan, China). Total RNA was extracted using AG RNAex Pro reagent (Accurate Biology) from U251, U87, and NHA. The Evo M-MLV RT Mix Tracking kit (Accurate Biology) was used for reverse transcriptase reaction, and SYBR-Green (Accurate Biology) was used for detection. The mRNA expression level of ARMC10, AUTS2, EN1, EREG, ERP29, HOXA2, HOXA5, HOXA7, HSPA5, LAP3, MDK, MTRF1L, NBEAL1, SLC6A6, and SLC37A3 was normalized by GAPDH. The primers of the fifteen genes were listed in **Supplementary Table S1**. Fold differences were calculated for each group using normalized CT values.

## Results

### Integrated analysis of single-cell transcriptomics and spatial transcriptomics of GBM

**Figure 1:**
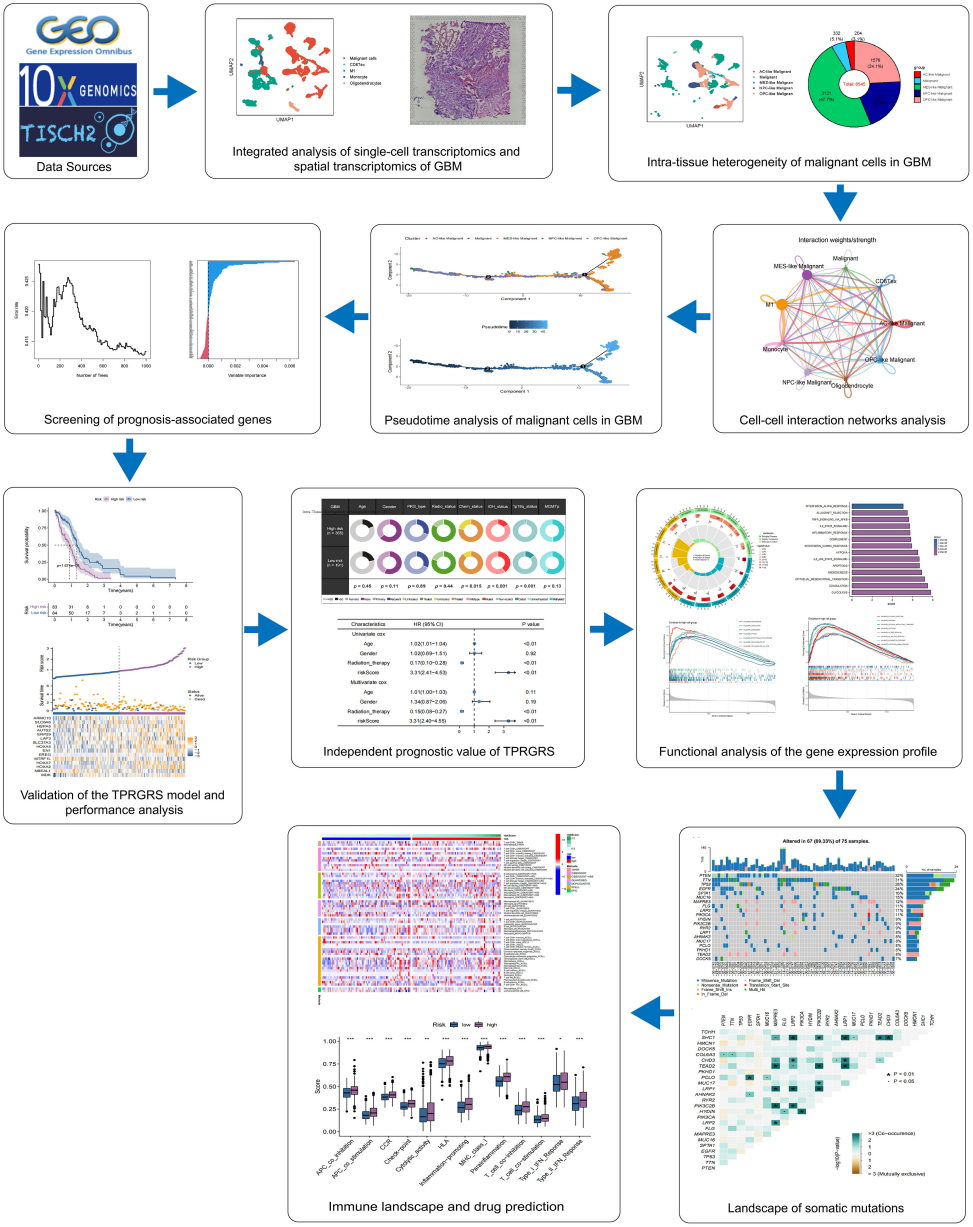
This study’s design and flowchart. *P < 0.05, ** P < 0.01, *** P < 0.001.

**Figure 2:**
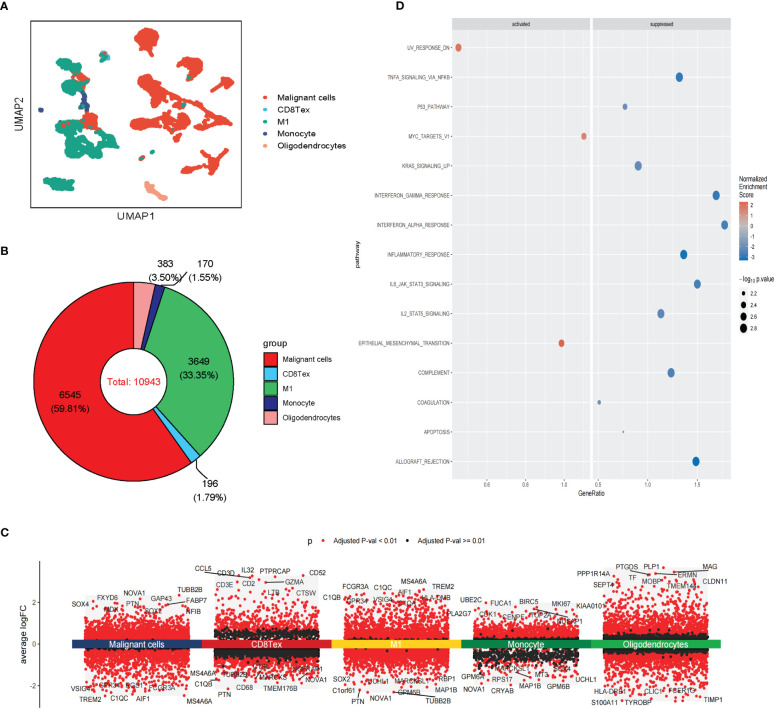
Single-cell RNA sequencing analysis of GBM. **(A)** The UMAP clustering map shows malignant cells, CD8 Tex, M1, Monocyte, and Oligodendrocytes representing the GBM cell types. **(B)** The proportion of malignant cells, CD8 Tex, M1, Monocyte, and Oligodendrocytes in GBM. **(C)** Differential gene expression analysis showing up- and downregulated genes across malignant cells, CD8 Tex, M1, Monocyte, and Oligodendrocytes. (Adjusted p-value: red for< 0.01, black for ≥ 0.01). **(D)** Bubble chart showing enrichment results of malignant cells based on GSEA analysis. (Normalized Enrichment Score: red for ≥ 0, blue for<0).

**Figure 1** presents the flowchart of our investigation. Based on the GBM scRNA-seq dataset (GBM_GSE131928_10x) collected by TISCH, we identified cell types by Seurat and used UMAP to visualize the dataset. The GBM scRNA-seq samples consisted of five cell types, including exhausted CD8 T cells (CD8 Tex), classically activated M1 macrophages (M1), Monocyte, Oligodendrocytes, and malignant cells (**Figure 2A**). We integrated the scRNA-seq and stRNA-seq data to construct the spatial map of these cells via CELLTREK (**Supplementary Figures S2A,B**). The spatial transcriptome showed the relative frequency of cells varies between glioblastoma samples, and malignant cells accounted for the highest proportion of cells in glioblastoma (**Figure 2B**; **Supplementary Figure S2C**). The proportion of M1 in the GBM was higher than in other non-malignant cells. Besides, Monocyte, Oligodendrocytes, and CD8Tex were less distributed in scRNA-seq or stRNA-seq samples. To summarize the cell spatial colocalizations in GBM, we applied the SColoc to the CellTrek result; M1 was identified as the hub and connected to the Monocyte, CD8Tex, and malignant cells (**Supplementary Figure S2D**).

Differentially expressed genes analysis of scRNA-seq analysis and stRNA-seq analysis is also employed (**Figure 2C**; **Supplementary Figure S2E**). The GSEA of malignant cells identified several common pathways across scRNA-seq. Strna-seq samples, such as UV response dn, Epithelial-mesenchymal transition (EMT), and MYC targets in Malignant cells, and pathways like Coagulation, Complement, Inflammatory response, Interferon alpha/gamma response, Interferon response, KRAS signaling up, IL2 STAT5, IL6 JAK STAT3 signaling, TNFA signaling via NFKB, Allograft rejection are activated in non-malignant cells (**Figure 2D**; **Supplementary Figure S2F**). These findings revealed research on glioblastoma in single-cell resolution is critical for the development of precision medicine.

### Intra-tissue heterogeneity of malignant cells

**Figure 3:**
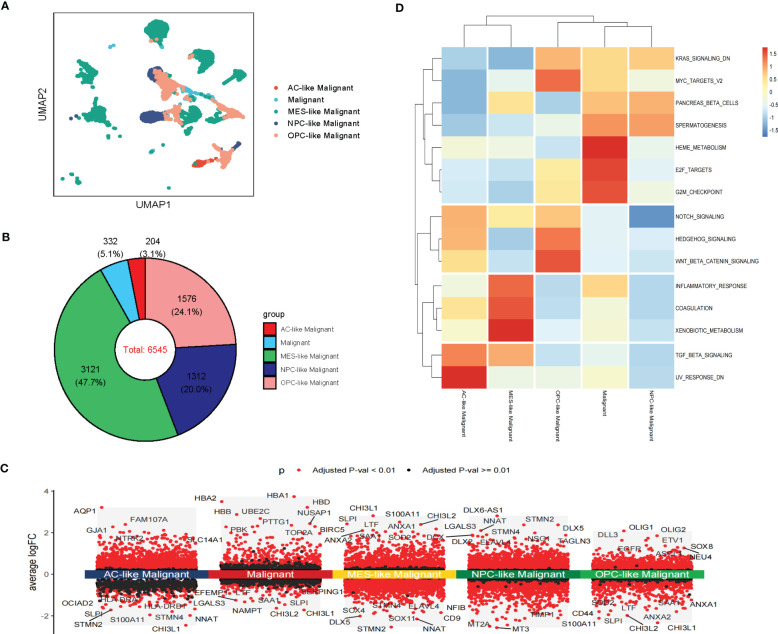
Intra-tissue heterogeneity of malignant cells in scRNA-seq. **(A)** The UMAP clustering map shows AC-like, MES-like, NPC-like, OPC-like, unclassified GBM malignant cells **(B)**The composition ratio of subtypes of malignant cells (AC-like, MES-like, NPC-like, OPC-like, and unclassified GBM malignant cells) in GBM patients. **(C)** Differential gene expression analysis showing up- and downregulated genes across subtypes of malignant cells (AC-like Malignant, MES-like Malignant, NPC-like Malignant, OPC-like Malignant, and unclassified GBM malignant cells). (Adjusted p-value: red for< 0.01, black for ≥ 0.01). **(D)** Various oncogenic hallmarks were enriched in AC-like Malignant, MES-like Malignant, NPC-like Malignant, OPC-like Malignant, and unclassified GBM malignant cells based on GSVA analysis.

We further analyzed the heterogeneity of malignant cells in glioblastoma. For the scRNA-seq data, we identified five clusters of malignant cells, including unclassified malignant cells and four subtypes of malignant cells: AC-like, MES-like, NPC-like, and OPC-like (**Figures 3A, B**). Then, we further revealed spatial patterns of subtypes of malignant cells (**Supplementary Figure S3A, B**). The density of MES-like Malignant cells was higher than other subtypes of malignant cells, suggesting that MES-like Malignant may be more aggressive than other subtypes in glioblastoma (**Figure 3B**; **Supplementary Figure S3C**). The cell spatial colocalizations of subtypes of malignant cells formed a linear graph. OPC-like Malignant cells were identified as the hub and connected to the other malignant cell subtypes except for MES-like Malignant cells (**Supplementary Figure S3D**).

Notably, these subtypes of malignant cells displayed transcriptional heterogeneity (**Figure 3C**; **Supplementary Figure S3E**). The GSVA indicated that unclassified malignant cells and four subtypes of malignant cells were enriched in different hallmarks in scRNA-seq data. Compared with other subtypes of malignant cells, Heme metabolism, E2F targets, G2M checkpoint, Pancreas beta cells, and Spermatogenesis were enriched in unclassified Malignant. TGF beta signaling and UV response dn were enriched in AC-like Malignant while coagulation, Inflammatory response, and Xenobiotic metabolism was enriched in MES-like Malignant. Notch signaling, Hedgehog signaling, Wnt beta-catenin signaling, KRAS signaling dn, and MYC targets v2 were enriched in OPC-like Malignant. PANCREAS beta cells, Spermatogenesis, and KRAS signaling dn were enriched in OPC-like Malignant. The results of GSVA analysis of stRNA-seq further revealed the heterogeneity of malignant cell subtypes (**Figure 3D**; **Supplementary Figure S3F**). Therefore, these subtypes of malignant cells may cause drug resistance in clinical treatment.

### Intercellular communications in the TME

**Figure 4:**
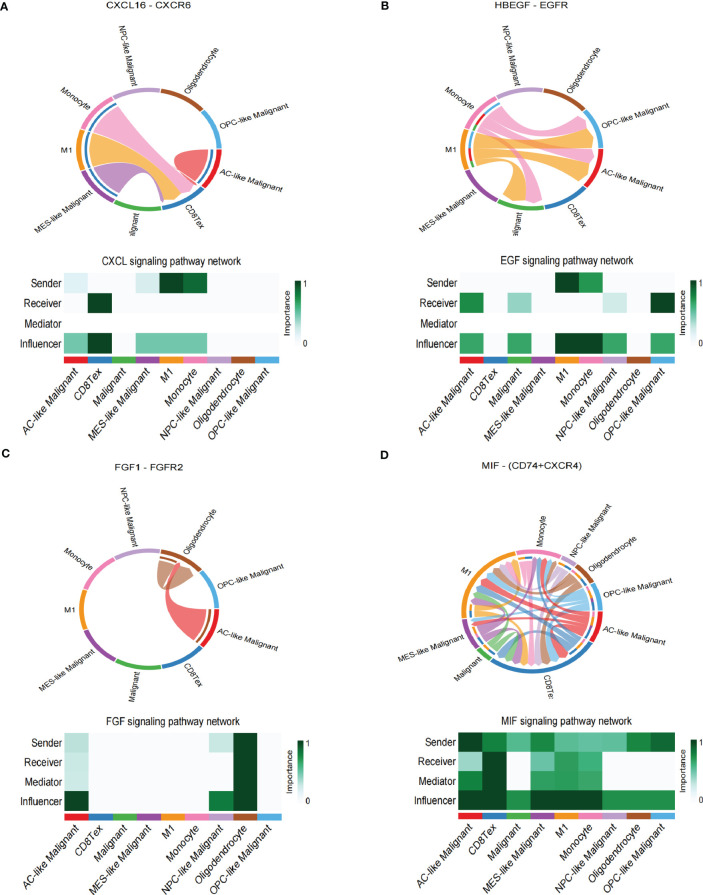
Cell-cell interaction networks of scRNA-seq. **(A)** CD8 Tex communicates with AC-like Malignant, MES-like Malignant, M1, and Monocyte through CXCL signaling. **(B)** AC-like Malignant, NPC-like Malignant, OPC-like Malignant, and unclassified malignant cells communicate with M1 and Monocytes through EGF signaling. **(C)** AC-like Malignant, NPC-like Malignant cells communicate with Oligodendrocytes through FGF signaling. **(D)**CD8 Tex, AC-like Malignant, MES-like Malignant cells, M1, and Monocyte interact through MIF signaling.

The spatial colocalization between different cell types reveals close contact between malignant and non-malignant cells in TME. Therefore, we investigated cell-cell communications in the TME through CellChat based on scRNA-seq analysis and identified several interactions in stRNA-seq analysis. In our work, CD8 Tex can receive the signal from MES-like Malignant, M1, and Monocyte through the CXCL signaling pathway (CXCL16-CXCR6, **Figure 4A**; **Supplementary S4A**). Monocytes can transmit signals to AC-like Malignant, OPC-like Malignant, and Malignant through the EGF signaling pathway (HBEGF-EGFR, **Figure 4B**; **Supplementary Figure S4B**). AC-like Malignant cells can communicate with Oligodendrocytes through the FGF signaling pathway (FGF1-FGFR2, **Figure 4C**; **Supplementary Figure S4C**). Furthermore, Malignant and four subtypes of Malignant cells: AC-like Malignant, MES-like Malignant, NPC-like Malignant, OPC-like Malignant communicate with CD8 Tex, M1, and Monocyte via MIF signaling pathway (MIF-(CD74+CXCR4), **Figure 4D**; **Supplementary Figure S4D**).

In summary, GBM cells communicate with Oligodendrocytes, CD8 Tex, M1, and Monocyte by comparing with the scRNA-seq sample and stRNA-seq sample may explain the immune landscape difference.

### Pseudotime analysis of malignant cells in GBM

**Figure 5:**
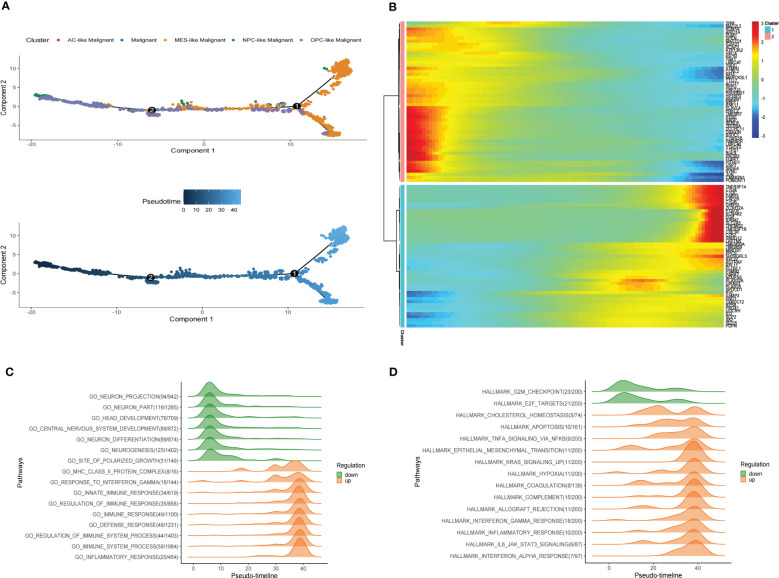
Pseudotime analysis of malignant cells in GBM scRNA-seq. **(A)** Pseudotime is shown in single-cell trajectories of subtypes of GBM malignant cells that monocle2 inferred. Pseudotime is shown in a gradient from dark blue to light blue, indicating the onset of pseudotime. **(B)**The Pseudotime heatmap shows gene expression dynamics of significantly labeled genes. Genes (rows) are clustered into two modules, and cells (columns) are sorted according to pseudotime. **(C)** Ridge plots show changes in biological processes along the pseudotime. **(D)**Ridge plots show changes in hallmarks along the pseudotime.

During the malignant transition of the tumor, malignant cells differentiate into different subtypes of cell populations due to their plasticity and the tumor environment (10, 11). To further explore the developmental trajectory of malignant cells, we performed the pseudotime analysis via Monocle2. The analysis suggested that the potential cell differentiation trajectories of the malignant cells: OPC-like and NPC-like Malignant cells are enriched at the root of the developmental trajectory, with MES-like Malignant cells at the terminus of the developmental trajectory (**Figure 5A**). Critically, we inferred the trajectories of malignant cells and mapped their pseudotime in stRNA-seq, and we observed a continuous spatial trajectory of the stRNA-seq spots (**Supplementary Figure S5A, B**).

We also identified differentially expressed genes of malignant cell subtypes that changed gene expression levels along the pseudotime. Significantly changed genes are shown as a heatmap in scRNA-seq (**Figure 5B**). To investigate pathways alterations during this transition, we used GeneSwitches to order pathways along the pseudotime-line. The altered biological processes showed that pathways along the pseudotime-line related to neuron development (such as neurogenesis, neuron differentiation, central nervous system development, head development, etc.) were downregulated at an early stage. Inflammation-related biological processes (such as inflammatory response, immune response, regulation of immune response, etc.) are upregulated later (**Figure 5C**). The top changed hallmarks along the pseudotime trajectory showed that hallmarks like G2M checkpoint and E2F targets downregulated at the early stage, while hallmarks such as Cholesterol homeostasis, EMT, Apoptosis, Hypoxia, TNFA signaling via NFKB, KRAS signaling upregulated later, Coagulation, Allograft rejection, Interferon alpha/gamma response, Inflammatory response, and IL6 JAK STAT3 signaling, which upregulated at the later stage (**Figure 5D**). Kaplan-Meier analysis of showed 5 of them such as Apoptosis, Coagulation, Hypoxia, EMT and IL6 JAK STAT3 signaling were associate with poor prognosis in GBM (**Supplementary Figure S6**) These results revealed the developmental trajectory of the malignant cells in GBM, and the transition of malignant cells may conduct variety clinical outcome.

### Establish tumor progress-related gene risk score

**Figure 6:**
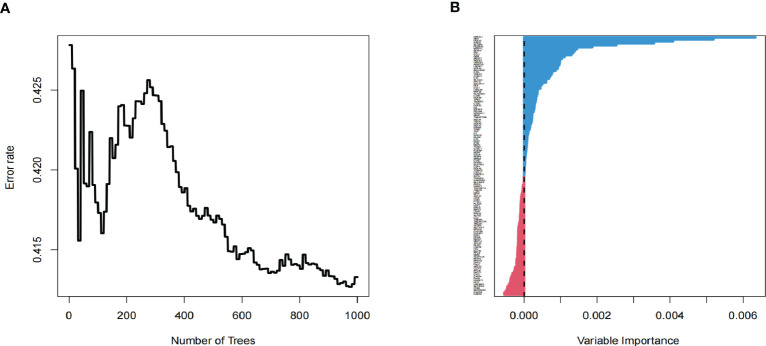
Screening of prognosis-associated genes. **(A)** Correlations between error rate and classification trees. **(B)** The relative importance of prognosis-associated genes.

**Figure 7:**
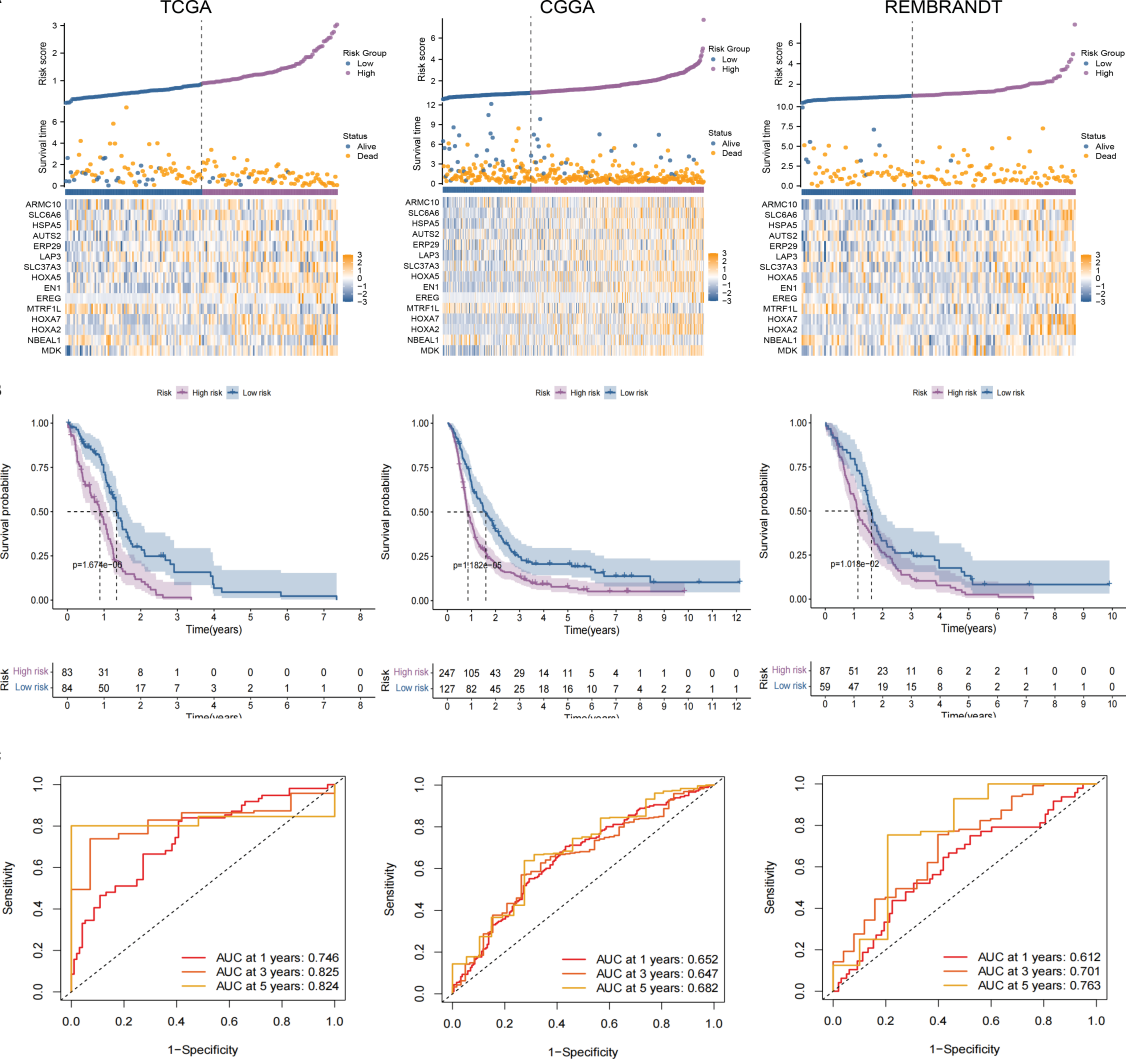
Validation of the TPRGRS model and performance analysis. **(A)** The overview of each patient’s survival status, risk rating distributions, and the expression of 15 TPRGRS genes in the TCGA dataset and two validation datasets (CGGA and REMBRANDT). **(B)** In three datasets, Kaplan-Meier survival curves revealed a shorter OS of the high-risk group than that of the low-risk group. **(C)** ROC curve analysis of the risk scores in three datasets, respectively.

As an essential component of the tumor microenvironment, the dynamics of malignant cells at the molecular and cellular levels significantly affect tumor development and metastasis. It is intriguing to identify the genes associated with the prognosis of patients with GBM from genes related to malignant cell differentiation. We performed univariable Cox analysis in the TCGA-GBM to screen for genes significantly associated with OS. Next, the RSF analysis and Multivariate Cox regression analysis were applied to screen 15 of them to construct the tumor progress-related gene risk score (TPRGRS), namely MDK, NBEAL1, HOXA2, HOXA7, MTRF1L, EREG, EN1, HOXA5, SLC37A3, LAP3, ERP29, AUTS2, HSPA5, SLC6A6 and ARMC10(**Figures 6A, B**). In the TCGA-GBM dataset, we constructed a risk score model including Expi and βi: $Risk\;score\;=\sum_{i=1}^{15}\left(Exp_{i}*\beta i\right)$ (**Supplementary Table S2**). GBM patients from TCGA-GBM (training dataset), CGGA (validation dataset 1), and REMBRANDT (validation dataset 2) were split into a high-risk group. A low-risk group based on the median value of the risk score, respectively (**Figure 7A**). The OS of the low-risk group was significantly longer than that of the high-risk group (**Figure 7B**). In addition, the ROC curve illustrated that TPRGRS was a remarkable prognostic predictor. For the TCGA-GBM training dataset, we analyzed this prognostic model’s 1-year, 3-year, and 5-year OS using ROC curves and obtained AUCs of 0.746, 0.825, and 0.824, respectively. Furthermore, ROC curve analysis on the CGGA and REMBRANDT validation datasets shows that TPRGRS had a favorable predictive performance (CGGA: AUC = 0.652 for 1-year, 0.647 for 3-year, 0.662 for 5-year survival; REMBRANDT: AUC = 0.612 for 1-year, 0.701 for 3-year, 0.763 for 5-year survival) (**Figure 7C**).

### Independent prognostic value of TPRGRS

**Figure 8:**
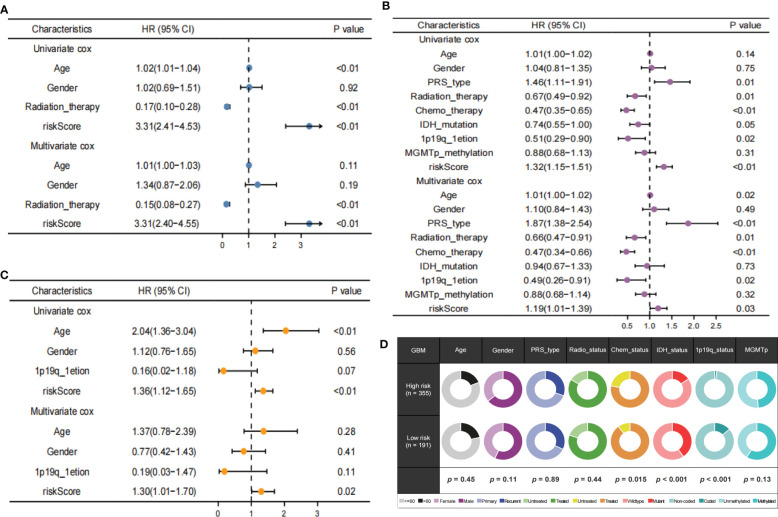
Independent prognostic value of TPRGRS. Analysis of the Univariate and Multivariate Cox regression analysis revealed risk score was strongly associated with OS in the TCGA training dataset **(A)**, the CGGA validation dataset **(B)**, and the REMBRANDT validation dataset **(C)**. Clinical traits analysis between the TPRGRS low-and high-risk groups **(D)**. Pair-wise Fisher’s Exact test p-values (*P< 0.01, ·P< 0.05).

Univariate analyses revealed that the clinical variables and risk scores were closely related to OS. Multivariate Cox analysis of the TCGA, CGGA and REMBRANDT datasets observed that risk score was an independent prognostic factor. (**Figures 8A–C**). Furthermore, the proportions of multiple clinical features in the high- and low-risk groups revealed that the IDH and 1p19q mutation status of GBM were significantly associated with risk score (**Figure 8D**). These results demonstrate that TPRGRS was a reliable signature associated with gene mutations and the prognosis of patients with GBM.

### Functional analysis and drug prediction of high-risk groups

**Figure 9:**
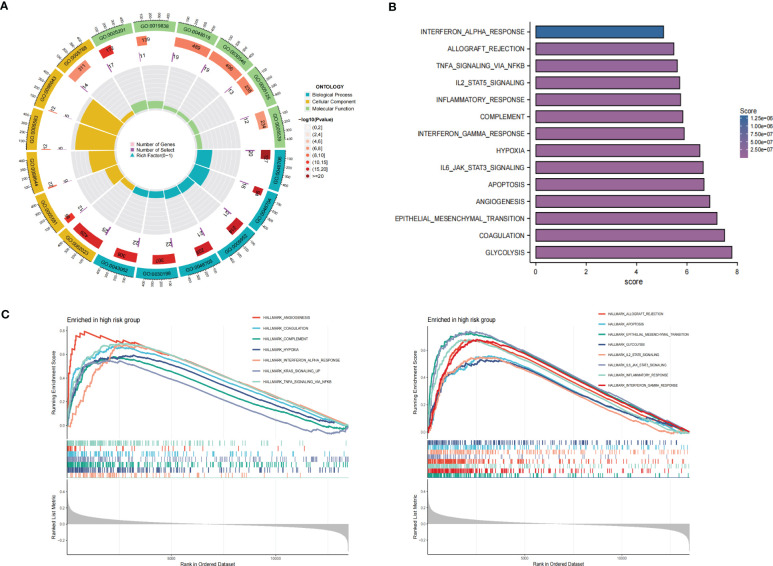
Functional analysis of the gene expression profile between the TPRGRS low- and high-risk groups. **(A)** GO enrichments analysis of upregulated genes in high-risk group in the TCGA dataset. **(B)** GSVA analysis showed enrichment scores of hallmark pathways in the high-risk group. **(C)** GSEA analysis reveals results for fifteen hallmark pathways associated with TPRGRS.

**Figure 10:**
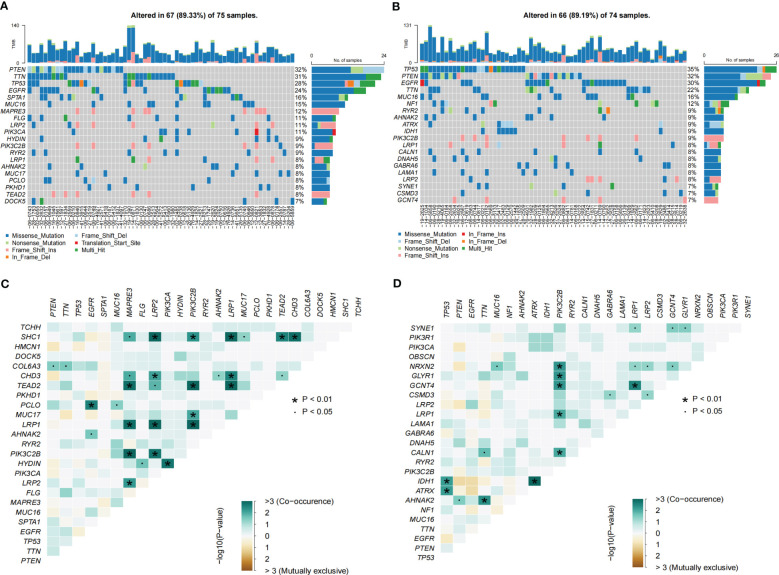
Landscape of somatic mutations in the TPRGRS low- and high-risk groups. **(A, B)** Waterfall plots displaying the somatic gene mutations of the high-**(A)** and low-**(B)** risk groups. Each column represents an individual patient. **(C, D)** The coincident and exclusive associations across mutated genes in high-**(C)** and low-**(D)** risk groups. The color or symbol of each cell represents the statistical significance of each pair of genes’ exclusivity or co-occurrence. Green represents mutual co-occurrence, and brown represents exclusive mutation. Pair-wise Fisher’s Exact test p-values (*P< 0.01, ·P< 0.05).

**Figure 11:**
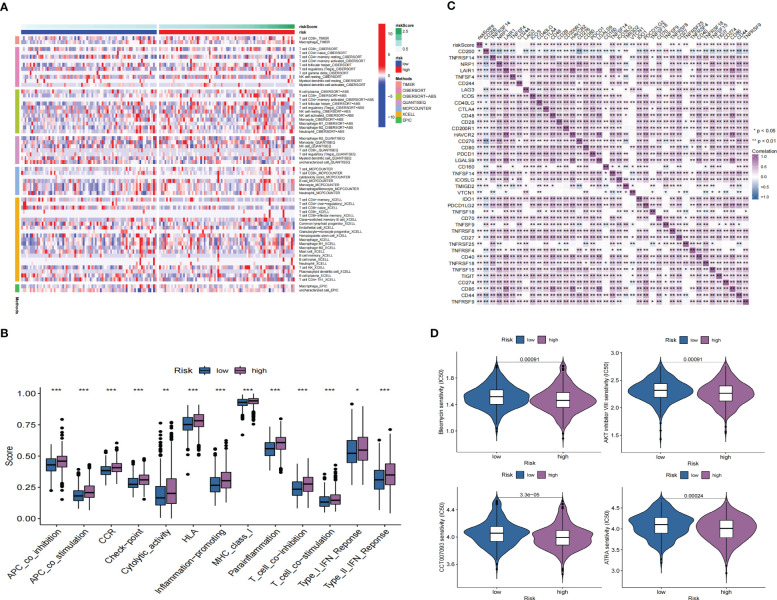
Immune landscape and drug prediction of high-risk groups. **(A)** The heatmap showing immune responses infiltration in the low- and high-risk groups. **(B)** ssGSEA showing higher enrichment scores of immunocytes-associated functions in the high-risk group. **(C)** A heatmap showing the differential expression of immune checkpoints in low- and high-risk groups. **(D)** The distribution of IC50 of four compounds of the TPRGRS low- and high-risk groups (*P< 0.05, ** P< 0.01, *** P < 0.001).

GO enrichment analysis of the DEGs between the low- and high-risk group elucidates that TPRGRS is mainly involved in growth factor binding, cytokine activity, and signaling receptor activator activity (**Figure 9A**; **Supplementary Table S3**). GSVA and GSEA analyses indicated that TPRGRS were associated with oncogenic hallmarks such as Angiogenesis, Complement, Glycolysis, EMT, Apoptosis, Hypoxia, TNFA signaling via NFKB, KRAS signaling up, Coagulation, Allograft rejection, Interferon alpha/gamma response, Inflammatory response, IL2 STAT5 and IL6 JAK STAT3 signaling (**Figures 9B, C**). Gene mutations between the high-risk patients and the low-risk patients showed that the top five genes with the highest mutation frequency in the high-risk group were PTEN, TTN, TP53, EGFR, and SPTA1, while the top five genes in the low-risk group were TP53, PTEN, EGFR, TTN, and MUC16 (**Figures 10A, B**). In addition, we analyzed the coincident and exclusive associations of the top 25 mutated genes from the high- (**Figure 10C**) and low- (**Figure 10D**) risk groups. Then, we explored the immune landscape of the low- and high-risk groups. The heatmap demonstrated that the abundances of CD4+ T cells, CD8+ T cells, neutrophils, and macrophages were markedly enriched in the high-risk group when compared to the low-risk group (**Figure 11A**). According to ssGSEA, immune functions, such as inflammation and HLA function, IFN response, markedly enriched in the high-risk group (**Figure 11B**). Immunosuppressive receptors (CTLA-4, LAG3, and TIGIT) and Immunosuppressive ligands (TNFSF14 and LGALS9) were significantly increased in the high-risk group (37–42) (**Figure 11C**). Further analysis predicted potential sensitive drugs for the high-risk group. AKT inhibitor VIII, ATRA, Bleomycin, and CCT007093 were identified as high-risk group-sensitive drugs (**Figure 11D**).

### External datasets and qRT-PCR validation

We validated the expression level of target genes from GEPIA. The results showed ARMC10, AUTS2, EN1, ERP29, HOXA2, HOXA5, HOXA7, HSPA5, LAP3, MDK, MTRF1L, NBEAL1, and SLC37A3 were significantly overexpressed in GBM compared with normal tissue (**Supplementary Figure S7A**). Moreover, qRT-PCR showed the expression of U251, U87, and NHA. The results showed that ARMC10, EREG, HOXA2, HOXA5, HSPA5, LAP3, MTRF1L, NBEAL1, and SLC37A3 were significantly upregulated in U251 and U87 cells compared with NHA cells (**Supplementary Figure S7B**).

## Discussion

The heterogeneity of glioblastoma is the main cause of treatment failure (43). Despite the heterogeneity in GBM patients, patients are treated according to clinical features and certain pathways, such as the P53 pathway (44). Moreover, therapeutic intervention may promote tumor progression by providing selective pressure to expand resistant subpopulations. Treatment of acquired drug-resistant tumors had to deal with heterogeneity. In the current study, we further analyzed GBM spatial and cellular heterogeneity by integrating analysis of scRNA-seq and stRNA-seq.

The immunocytes infiltration ratio between scRNA-seq and stRNA-seq GBM samples revealed that GBM samples are lymphocyte-depleted subtypes. In our study, functionally exhausted CD8T cells with a low infiltration ratio in the TME indicated T-cell dysfunction in the glioblastoma microenvironment. Recent studies have reported that M1 could recruit and modify the activation of endogenous macrophages and NK cells to regulate the immune microenvironment (45). The spatial colocalization of stRNA-seq data confirmed the close contact between M1 and malignant cells. These findings suggest that M1-based immunotherapy has excellent potential in GBM.

At the single-cell level, the GSEA of malignant cells revealed some biological characteristics, which included UV response dn, EMT, and MYC targets. Then, we identified unclassified malignant cells and four subtypes of malignant cells (MES-like, AC-like, OPC-like, and NPC-like) in scRNA-seq and stRNA-seq. Previous studies described proneural subtypes (OPC-like Malignant and NPC-like Malignant) relating to a more favorable outcome, while MES-like Malignant related to poor survival (46). In the current study, MES-like Malignant was identified as a prominent type in scRNA-seq or stRNA-seq GBM samples. The spatial distribution of MES-like Malignant showed more aggression than other malignant subtypes. Our work further explored the heterogeneity of their spatial component in stRNA-seq analysis. Spatial colocalization analysis of malignant subtypes uncovered the connection of the subtypes in spatial pattern, and OPC-like Malignant was identified hub that connects other malignant cells. Notably, these malignant cell subtypes revealed transcriptional heterogeneity. The functional enrichment results varied significantly among the malignant subtypes. Some famous oncogenic hallmarks, such as P53 signaling and Apoptosis pathways, are not enriched in malignant cells, implying that the heterogeneity of malignant cells is an obstacle to drug or immunotherapy treatments.

Additionally, spatial colocalization confirmed the close contact between these malignant and non-malignant cells. Thus, cell interactions between GBM cells and immunocytes are crucial for further research in GBM immune landscape. Then, cell chat was applied to predict cell-cell communications between malignant and non-malignant cells to explore their potential mechanism in scRNA-seq. We further identified these ligand-receptor pairs of pathways in stRNA-seq. Their roles in mediating GBM cell progression have been proved, but their connection with immunocytes is elusive. Recent studies demonstrated that CXCL16 signaling is a target to modulate macrophage phenotype to restrain inflammation and limit glioma progression (47). Oligodendrocytes can express the FGF1 induce stemness and chemo-radio resistance in GBM cells (48). HBEGF is expressed in monocytes that can stimulate EGFR on malignant cells to promote tumor progression and metastasis (49). MIF and its receptors CD74 and CXCR4 were identified as potential targets for GBM treatment (50). In conclusion, GBM cells and the tumor microenvironment create a milieu that ultimately promotes tumor cell transcriptomic adaptability and disease progression.

Malignant cells differentiate into different subpopulations due to their plasticity and the tumor environment (10, 11). The transition of GBM cells from proneural to mesenchymal was associated with treatment resistance in GBM relapse (51, 52). Thus, we applied pseudotime analysis to construct the potential developmental trajectory of malignant cells and spatial mapping of the pseudotime values in the tissue section, heterogeneity in the proneural–mesenchymal transition. The gene expression level varies during pseudotime, partially due to the transcriptomic adaptability of the transition of GBM cells from proneural to mesenchymal. Altered biological processes and oncogenic hallmarks during this transition revealed that MES-like Malignant showed more aggression than other subtypes. Critically, we also noticed pathways such as Angiogenesis, Hypoxia, EMT, and IL6 JAK STAT3 signaling changed during this transition, which was associated with poor prognoses in GBM. These results indicate the transition of GBM cells caused a variety of clinical outcomes.

Thus, it is essential to identify genes involving malignant cell differentiation and the prognosis of GBM. Currently, there are many algorithms to construct genetic risk models, such as the least absolute shrinkage and selection operator (LASSO) or Random Forest. Their models can predict the survival situation (53–56). Unlike other algorithms, Random Forest enables relatively easy estimations of variable importance and minimizing overfitting (57, 58). Therefore, this study is based on Random Forest to build a genetic risk model for patients with GBM.

Previous studies constructed gene signatures for GBM to predict the prognosis of GBM patients. Wang et al. established a prognostic model for four autophagy-related genes (59). Li et al. constructed a prognostic model for three pyroptosis-related genes and hypoxia phenotype-related gene signatures, respectively (60). Most of them were constructed based on bulkRNA-seq, which ignores heterogeneity in GBM samples. Genes of these signatures expressed related to the malignant cell are unknown in the present study, and we identified genes associated with malignant cell differentiation according to pseudotime analysis. Next, we applied Cox regression, random forest, and Kaplan-Meier analyses to screen prognosis-associated mRNAs further. Finally, 15 genes were selected to construct TPRGRS to predict the prognosis for GBM. TPRGRS is an independent prognostic factor compared with other clinical features, which is associated with the overall survival status of the TCGA GBM dataset. Prognosis analysis validated the predictive performance of TPRGRS in two external datasets (CGGA and REMBRANDT).Unlike PRS type and other clinical factors of GBM that are not correlated with TPRGRS, abnormalities in GBM such as mutations in IDH and 1p19q which contribute to the heterogenety of tumor were significantly associated with TPRGRS (61, 62).

In our study, genes of TPRGRS were identified to be associated with prognosis in GBM, namely MDK, NBEAL1, HOXA2, HOXA7, MTRF1L, EREG, EN1, HOXA5, SLC37A3, LAP3, ERP29, AUTS2, HSPA5, SLC6A6, and ARMC10. Some of them have already been reported. MDK can promote GBM cell proliferation, angiogenesis, anti-apoptotic activity, and induce multidrug resistance (63). NBEAL1 was identified as upregulated in glioma (64). Hox genes (HOXA2, HOXA5, and HOXA7) can regulate several hallmarks of cancer in malignant glial tumors (65). EREG promotes tumor progression via EREG/EGFR pathway (66). In addition, AUTS2, EN1, and HSPA5 are involved in regulating nervous system maturation (67–69). LAP3 could promote migration and invasion of glioma cells (70). ERP29 was identified as a prognostic marker and suppressor in tumors (71). SLC6A6 contributes to the 5-aminolevulinic acid (ALA)-induced accumulation in the signal transmission process of the nervous system (72). ARMC10 plays a role in cell growth and survival via regulations of mitochondrial dynamics (73). Additionally, some novel molecules identified as novel prognostic markers were first proposed in this work, including SLC37A3 and MTRF1L, which can be served as predictors for GBM patient prognosis.

GO analysis revealed genes of TPRGRS were related to growth factor binding, cytokine activity, and signaling receptor activator activity functions. GSVA and GSEA analysis further confirmed TPRGRS were significantly associated with oncogenic hallmarks such as Angiogenesis, Glycolysis, EMT, Hypoxia, Inflammatory response, etc. Besides, gene mutation analysis in GBM was essential for chemotherapy and immunotherapy. We found that most of the mutations in TPRGRS-related signature genes were PTEN, and its mutation enhanced the invasiveness of GBM (74). Furthermore, the analysis of immune cell infiltration showed that the infiltration of immune cells and immune functions in the high-risk group were relatively active compared to those in the low-risk group. Notably, the infiltration of macrophages and CD8+ T cells was significantly higher in the high-risk group than in the low-risk group. These cells could contradict an antitumor effect via CXCL16-CXCR6 signaling (47, 75). On this basis, the expression of immunosuppressive receptors (CTLA4, LAG3, and TIGIT) and immunosuppressive ligands (LGALS9 and TNFSF14) were identified as higher in the high-risk group. These molecules are associated with inefficient control of cancers and promote immune tolerance in GBM (76). Understanding the molecular mechanism of immune checkpoints could also contribute to immunotherapeutic interventions. TPRGRS may provide new insights for predicting the immune landscape in GBM patients.

In addition, potential targeted drugs for high-risk score samples are predicted through the CGP database: AKT inhibitor VIII, ATRA, Bleomycin, and CCT007093. AKT inhibitor VIII is a selective inhibitor of Akt1, Akt2, and Akt3, significantly enhancing the antiproliferative capacity of fluprednidenes (77). ATRA, with extensive antiproliferative and pro-differentiation activity capabilities, can inhibit the malignant growth of various tumor cells and enhance the therapeutic effect of radiotherapy (78). Bleomycin and CCT007093 are also commonly used for cancer treatments that can inhibit tumor progression (79). Thus, the predictive performance of TPRGRS was confirmed as a predictor for GBM treatment chemotherapy, and choosing drugs based on TPRGRS may contribute to the precision medicine of GBM.

However, several limitations ought to be pointed out in our research. First, we collected the scRNA-seq and stRNA-seq data from different patients. Thus, it is limited to identifying cells of stRNA-seq data. Second, the TPRGRS was constructed based on retrospective analysis and lacked some clinical information. Third, the current study needs further experiments *in vivo* or *in vitro* to investigate the specific mechanism of genes of TPRGRS.

In conclusion, we provide new insights into GBM through integrated analysis of scRNA-seq and stRNA-seq data. Our results revealed the spatial TME and heterogeneity in malignant cells. Heterogeneity during the proneural–mesenchymal transition of GBM was analyzed by constructing the differentiation trajectory of subtypes of malignant cells as well. We constructed a prognostic model based on integrated analysis of bulkRNA-seq and scRNA-seq data, which can predict the overall survival of GBM patients. The TPRGRS, in combination with routine clinicopathological evaluation of tumors, may provide more personalized drug regimens for GBM patients.

## Data availability statement

The datasets presented in this study can be found in online repositories. The names of the repository/repositories and accession number(s) can be found in the article/**Supplementary material**.

## Ethics statement

Ethical review and approval were not required for the study on human participants following the local legislation and institutional requirements. This study did not require informed consent for participation following the national legislation and institutional requirements.

## Author contributions

BD and CL conceived the idea and supervised the overall project. YL, ZW, and YF analyzed the data and wrote the manuscript. JG and BW participated in the revision of the manuscript. All authors read and approved the final manuscript.
