# Supplementary material for: Integration analysis of single-cell and spatial transcriptomics reveal the cellular heterogeneity landscape in glioblastoma and establish a polygenic risk model
Source: Front Oncol. 2023 Jun 15;13:1109037. doi: 10.3389/fonc.2023.1109037 (PMC10308022; doi:10.3389/fonc.2023.1109037)
Supplement: Supplementary file 1 [file DataSheet_1.pdf]

Supplementary Material

Supplementary Table S1. The primer sequences of TPRGRS mRNAs

*Sequence of primers(5'-3')	Name of primer
ACAACTTTGGTATCGTGGAAGG	F-GAPDH
GCCATCACGCCACAGTTTC	R-GAPDH
CGCGGTCGCCAAAAAGAAAG	F-MDK
TACTTGCAGTCGGCTCCAAAC	R-MDK
TATGCCTCCAGGAATATCTCTGC	F-NBEAL1
ATGTACGAGCAAGTCCCAATTT	R-NBEAL1
ACCCCGAAGGGTGGAGATT	F-HOXA2
CGGAGTCCTCAAGGCTTTTACAT	R-HOXA2
CGTTCCGGGCTTATACAATGT	F-HOXA7
CTCGTCCGTCTTGTCGCAG	R-HOXA7
CAAGTGAACTAGGTGGCCTTAG	F-MTRF1L
TGTCTTTGGCACTCTTTGTACTC	R-MTRF1L
ATGTGGCTTTGACCGTGATTC	F-EREG
TCCCCTGAGGTAACTCTCTCATA	R-EREG
CGCAGCAGCCTCTCGTATG	F-EN1
CCTGGAACTCCGCCTTGAG	R-EN1
AACTCATTTTGCGGTCGCTAT	F-HOXA5
TCCCTGAATTGCTCGCTCAC	R-HOXA5
GTGCTTTTAACACGTCAGTTGAG	F-SLC37A3
GCCCACAGCATAGGAGAAGAG	R-SLC37A3
TCGGCAAAGCTCTATGGAAGT	F-LAP3
GCGTCATCTCATTGGCTGG	R-LAP3
GGGGCAGTTAAGGTTGGAGC	F-ERp28
TCTCCTTCACACTTGAGAGGTT	R-ERp28
GGGCTCCGACAAGGAAGAC	F-AUTS2
TGGCGTTTCTCCACACGTTC	R-AUTS2
GAAAGAAGGTTACCCATGCAGT	F-HSPA5
CAGGCCATAAGCAATAGCAGC	R-HSPA5
GGAGAAGTGGTCTAGCAAGATCG	F-SLC6A6
AGAAACGCACCTCCACCATTC	R-SLC6A6
CTGGCTGGACTGACATTGTTG	F-ARMC10

TGCACCTTCGTGTTTCCATTT	R-ARMC10

Supplementary Table S2. The prognostic significance of the 15-genes signature.

Gene	Coef
MDK	0.080553462
NBEAL1	-0.463518995
HOXA2	0.354758184
HOXA7	0.181111051
MTRF1L	-0.50220884
EREG	0.345837887
EN1	0.091256054
HOXA5	-0.268859218
SLC37A3	0.179417566
LAP3	0.162479147
ERP29	0.252448906
AUTS2	0.203477645
HSPA5	-0.207701827
SLC6A6	0.299355362

Supplementary Table S3. Gene ontology enrichment analysis of differentially expressed genes

ID	Description
GO:0048706	embryonic skeletal system development
GO:0048704	embryonic skeletal system morphogenesis
GO:0009952	anterior/posterior pattern specification
GO:0048705	skeletal system morphogenesis
GO:0030198	extracellular matrix organization
GO:0062023	collagen-containing extracellular matrix
GO:0005581	collagen trimer
GO:0098644	complex of collagen trimers
GO:0005583	fibrillar collagen trimer
GO:0098643	banded collagen fibril
GO:0005201	extracellular matrix structural constituent
GO:0019838	growth factor binding
GO:0048018	receptor ligand activity
GO:0030546	signaling receptor activator activity
GO:0005125	cytokine activity

Supplementary Figure S1

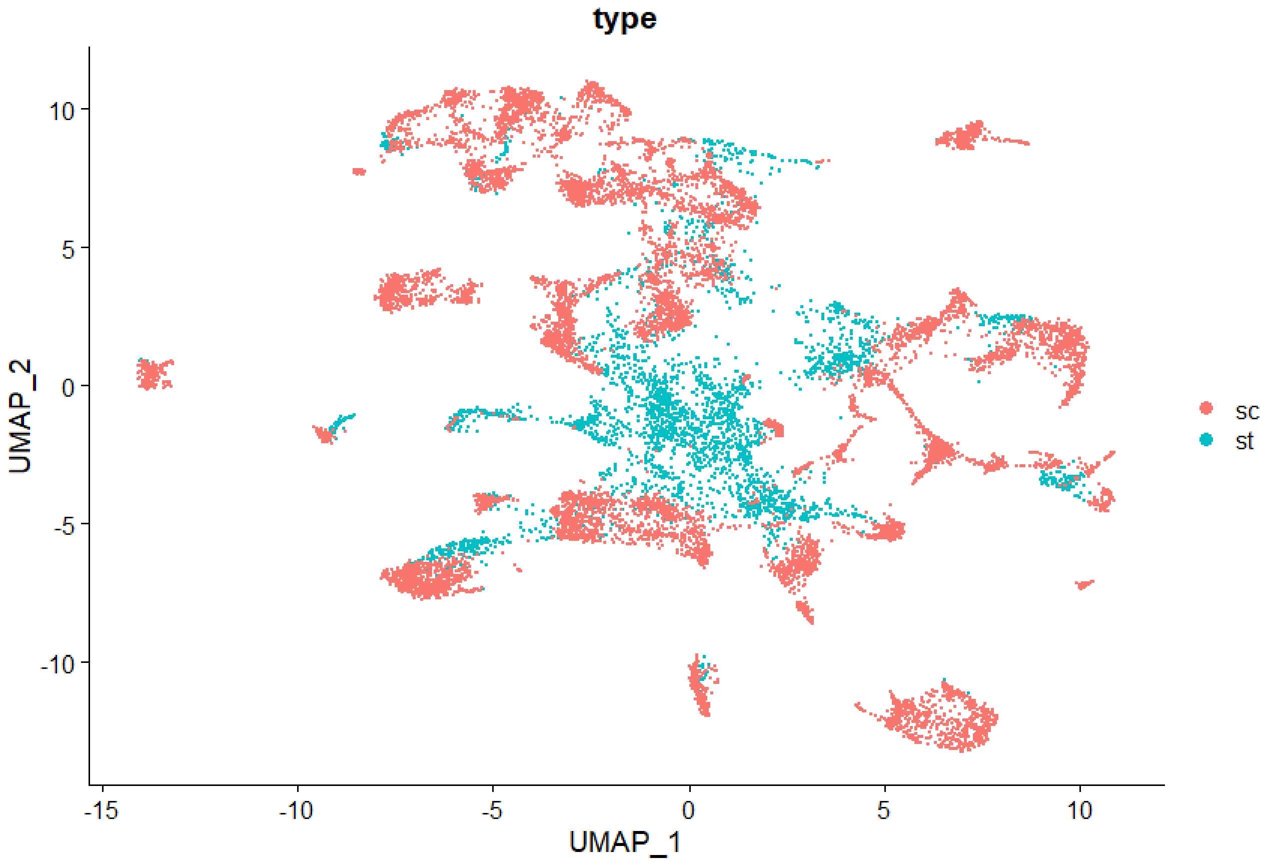

Figure. S1. UMAP shows the co-embedding result of stRNA-seq and scRNA-seq datasets using the CELLTREK.

Supplementary Figure S2

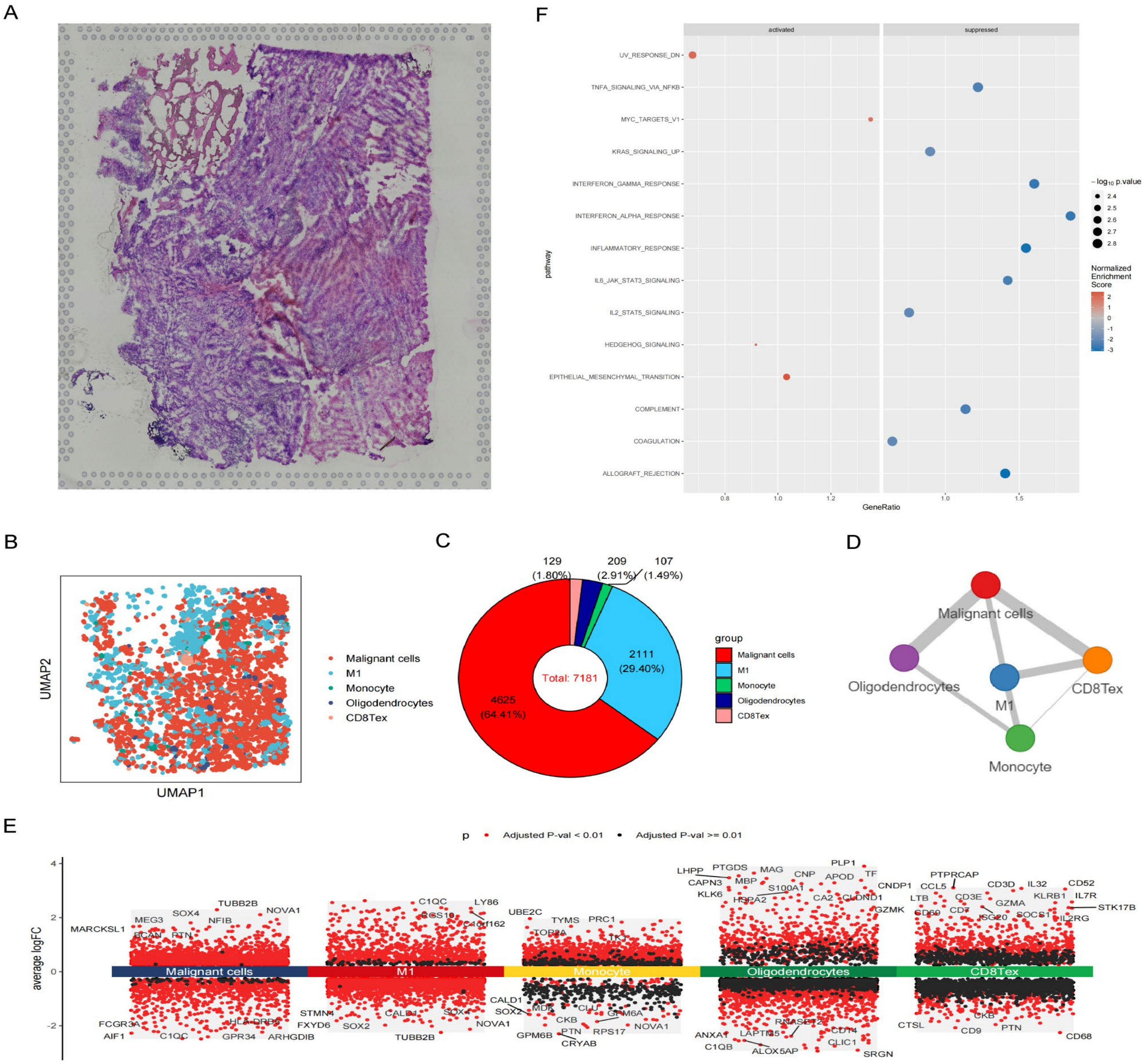

Figure. S2. Spatial tumor microenvironment of GBM. (A) The hematoxylin and eosin (H&E) image of GBM tissue.(B)CellTrek spatial mapping of malignant cells, CD8 Tex, M1, Monocyte, and Oligodendrocytes representing the GBM cell types in stRNA-seq. (C) The relative proportion of malignant cells, CD8 Tex, M1, Monocyte, and Oligodendrocytes in stRNA-seq. (D) Spatial colocalization graph of malignant cells, CD8 Tex, M1, Monocyte, and Oligodendrocytes using SColoc. (E) Differential gene expression analysis showing up- and downregulated genes across malignant cells, CD8 Tex, M1, Monocyte, and Oligodendrocytes in stRNA-seq. Adjusted p-val: red for < 0.01, black for ≥ 0.01. (F) Bubble chart showing enrichment results of malignant cell based on GSEA analysis in stRNA-seq. Normalized Enrichment Score: red for ≥ 0, blue for <0.

Supplementary Figure S3

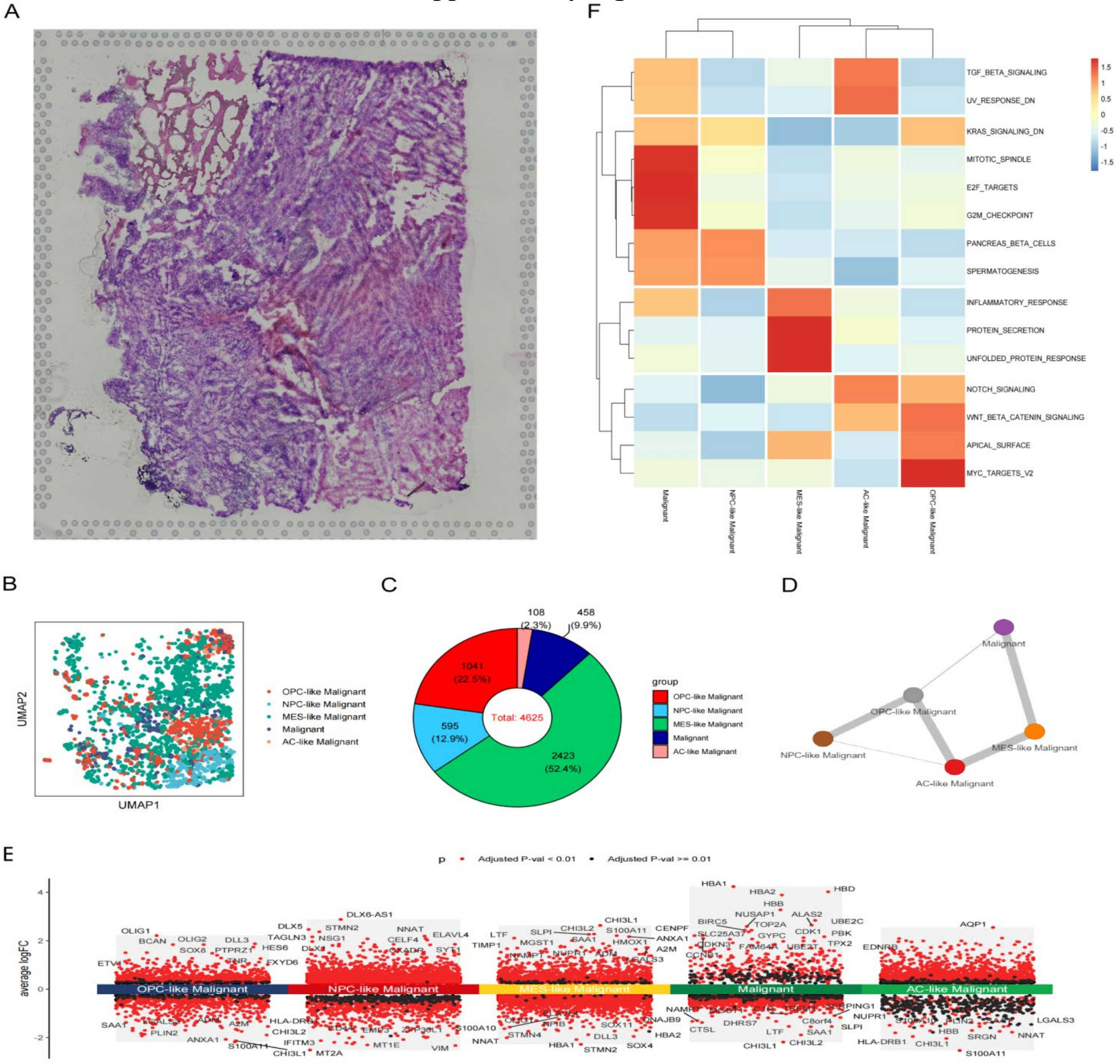

Figure. S3. Heterogeneity of Malignant Cells in stRNA-seq.(A) The hematoxylin and eosin (H&E) image of GBM tissue.(B) Spatial cell charting of malignant subtypes using CellTrek. (C) The relative proportion of GBM malignant subtypes. (D)Spatial colocalization graph of malignant subtypes. (E) Differential gene expression analysis showing up- and downregulated genes across subtypes of malignant cells in stRNA-seq (AC-like Malignant, MES-like Malignant, NPC-like Malignant, OPC-like Malignant, and unclassified GBM malignant cells). Adjusted p-val: red for < 0.01, black for ≥ 0.01. (F) Various hallmark gene sets were enriched in Various oncogenic hallmarks were enriched in AC-like Malignant, MES-like Malignant, NPC-like Malignant, OPC-like Malignant, and unclassified GBM malignant cells in stRNA-seq

Supplementary Figure S4

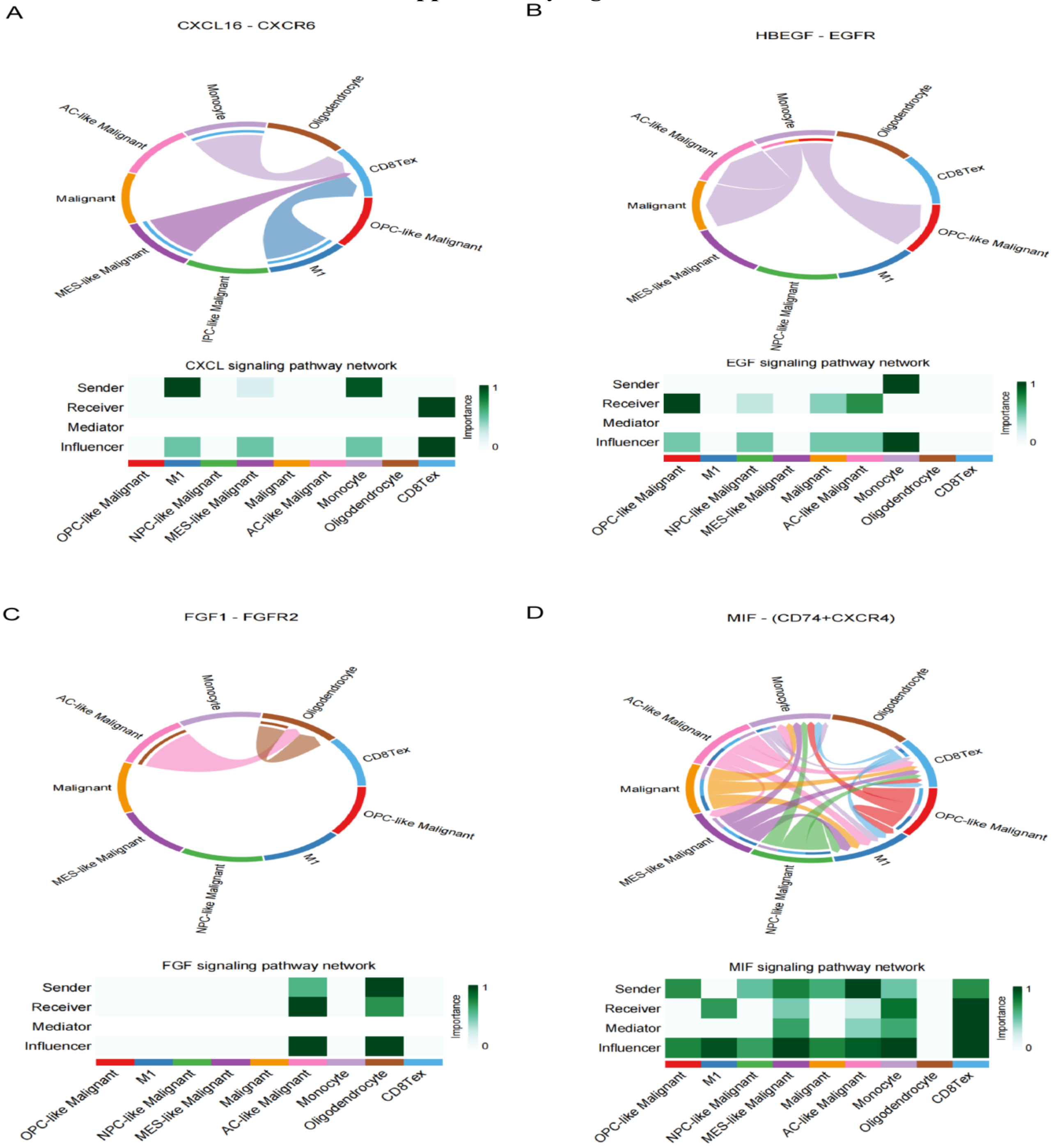

Figure. S4. Cell-cell interaction networks of stRNA-seq.(A) CD8 Tex communicates with MES-like Malignant, M1, and Monocyte through CXCL signaling. (B) AC-like Malignant communicate with Oligodendrocytes through FGF signaling. (C) AC-like Malignant, NPC-like Malignant, OPC-like Malignant and unclassified malignant cells communicate with Monocyte through EGF signaling. (D) CD8 Tex, M1, Monocyte, AC-like Malignant, MES-like Malignant, NPC-like Malignant, OPC-like Malignant, and unclassified malignant cells interact with each other through MIF signaling.

Supplementary Figure S5

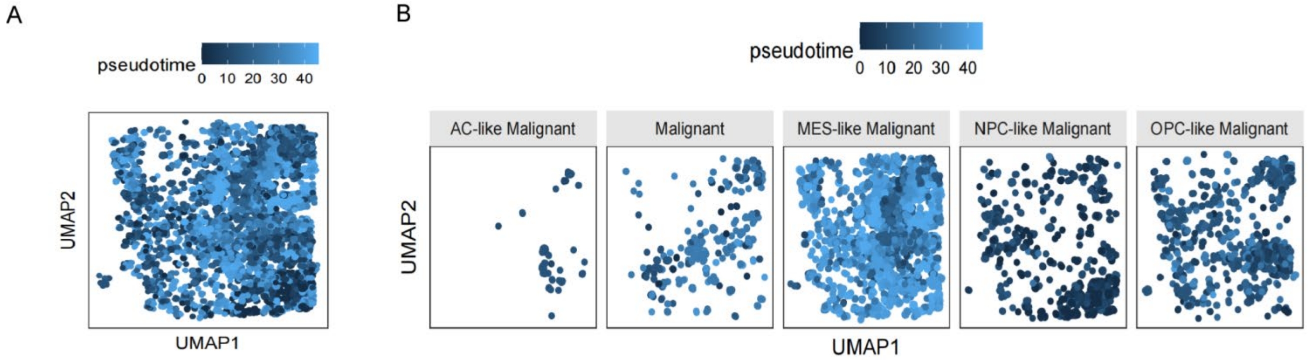

Supplementary FigureS5. Pseudotime values of malignant cells in GBM stRNA-seq.(A)Spatial mapping of the pseudotime values of trajectory analysis of GBM malignant cells in the scRNA-seq. (B)AC-like Malignant, MES-like Malignant, NPC-like Malignant, OPC-like Malignant, and unclassified GBM malignant cells are plotted with pseudotime values in spatial spots.

Supplementary Figure S6

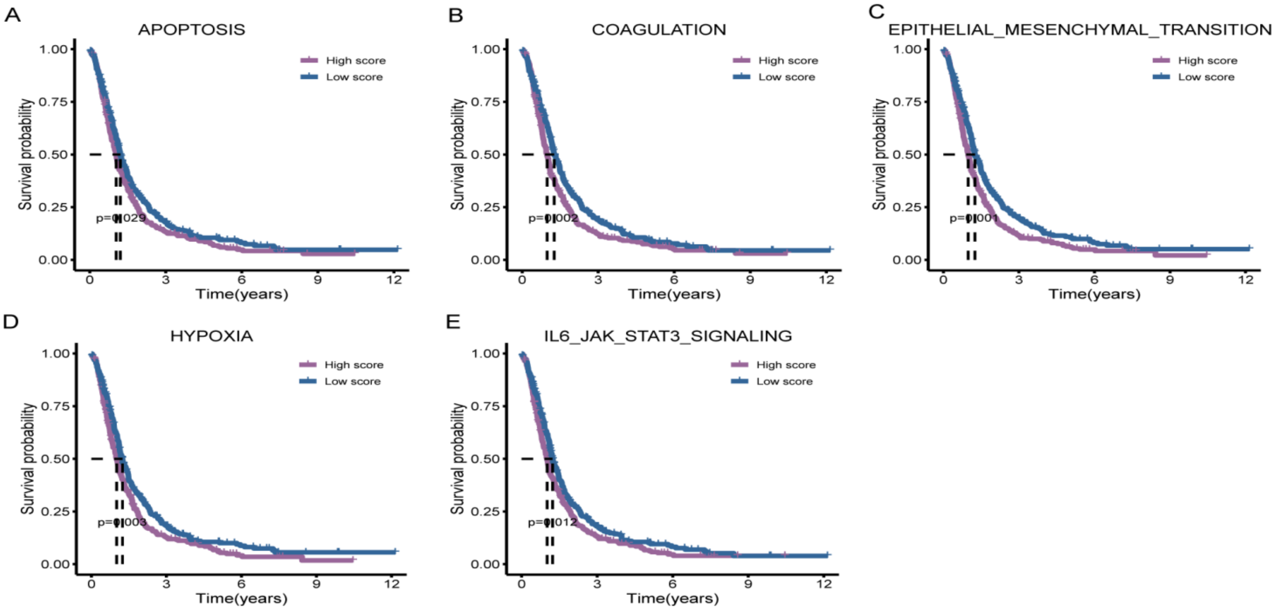

Figure. S6. Kaplan-Meier analysis revealed high enrichment scores of Apoptosis(A), Coagulation(B), EMT(C), Hypoxia(D), and IL6 JAK STAT3 signaling (E) are associated with shorter OS in GBM patients.

Supplementary Figure S7

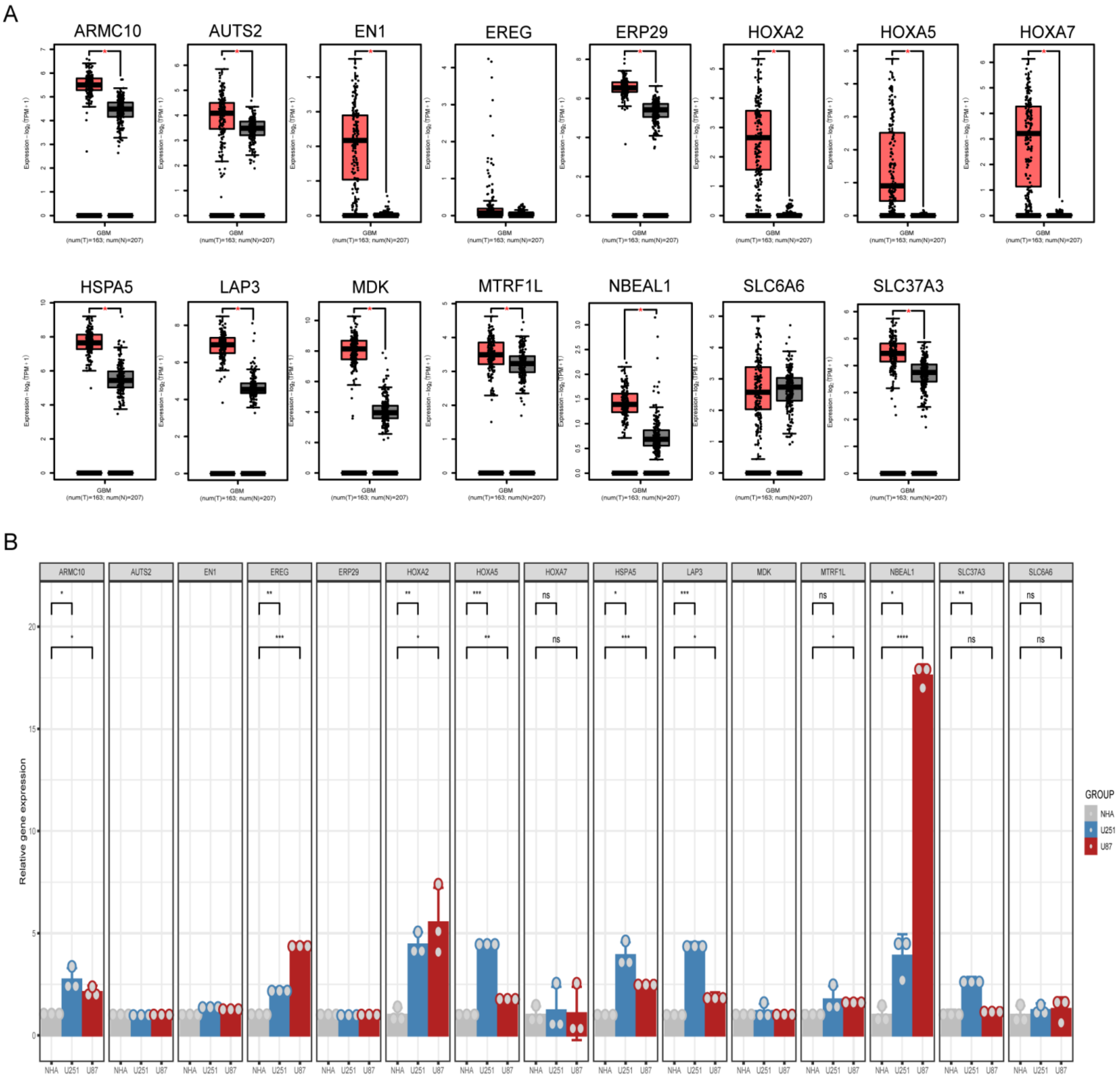

Supplementary Figure S7. The expression profile of the TPRGRS with external validation

(A)Individual expression profiles of the target genes in GEPIA. (B) The gene expression level in glioma cells and normal human astrocytes. *p < 0.05; **p < 0.01; ***p < 0.001.
